# TRIM33 Overexpression Inhibits the Progression of Clear Cell Renal Cell Carcinoma In Vivo and In Vitro

**DOI:** 10.1155/2020/8409239

**Published:** 2020-08-25

**Authors:** Yingkun Xu, Guangzhen Wu, Jiayao Zhang, Jianyi Li, Ningke Ruan, Jianfeng Zhang, Zhiyu Zhang, Yougen Chen, Qi Zhang, Qinghua Xia

**Affiliations:** ^1^Department of Urology, Shandong Provincial Hospital, Cheeloo College of Medicine, Shandong University, Jinan, Shandong 250021, China; ^2^Department of Urology, The First Affiliated Hospital of Dalian Medical University, Dalian, Liaoning 116011, China; ^3^Liver Transplantation Center and Hepatobiliary Surgery, Shandong Provincial Hospital, Cheeloo College of Medicine, Shandong University, Jinan, Shandong 250021, China; ^4^The Nursing College of Zhengzhou University, Zhengzhou, Henan 450001, China; ^5^Department of Surgical Oncology and General Surgery, Key Laboratory of Precision Diagnosis and Treatment of Gastrointestinal Tumors, Ministry of Education, The First Affiliated Hospital of China Medical University, Shenyang, Liaoning 110122, China; ^6^Department of Urology, The Affiliated Yantai Yuhuangding Hospital of Qingdao University, Yantai, Shandong 264000, China; ^7^Department of Urology, Shandong Provincial Hospital Affiliated to Shandong First Medical University, Jinan, Shandong 250021, China

## Abstract

**Purpose:**

To evaluate the expression of tripartite motif-containing 33 (TRIM33) in ccRCC tissues and explore the biological effect of TRIM33 on the progress of ccRCC.

**Method:**

The Cancer Genome Atlas (TCGA) database was used to examine the mRNA expression levels of TRIM33 in ccRCC tissues and its clinical relevance. Immunohistochemistry (IHC) was performed to evaluate its expression in ccRCC tissues obtained from our hospital. The correlation between TRIM33 expression and clinicopathological features of the patients was also investigated. The effects of TRIM33 on the proliferation of ccRCC cells were examined using the CCK-8 and colony formation assays. The effects of TRIM33 on the migration and invasion of ccRCC cells were explored through wound healing and transwell assays, along with the use of Wnt signaling pathway agonists in rescue experiments. Western blotting was used to explore the potential mechanism of TRIM33 in renal cancer cells. A xenograft model was used to explore the effect of TRIM33 on tumor growth.

**Result:**

Bioinformatics analysis showed that TRIM33 mRNA expression in ccRCC tissues was downregulated, and low TRIM33 expression was related to poor prognosis in ccRCC patients. In agreement with this, low TRIM33 expression was detected in human ccRCC tissues. TRIM33 expression levels were correlated with clinical characteristics, including tumor size and Furman's grade. Furthermore, TRIM33 overexpression inhibited proliferation, migration, and invasion of 786-O and ACHN cell lines. The rescue experiment showed that the originally inhibited migration and invasion capabilities were restored. TRIM33 overexpression reduced the expression levels of *β*-catenin, cyclin D1, and c-myc, and inhibited tumor growth in ccRCC cells in vivo.

**Conclusion:**

TRIM33 exhibits an abnormally low expression in human ccRCC tissues. TRIM33 may serve as a potential therapeutic target and prognostic marker for ccRCC.

## 1. Introduction

According to 2018 global cancer statistics, renal cell carcinoma (RCC) is one of the most common cancers in the world, with more than 400,000 patients newly diagnosed, and more than 170,000 deaths due to RCC each year [1]. Among them, clear cell renal cell carcinoma (ccRCC) is the most common RCC subtype, accounting for 75% of all primary kidney cancers [2]. ccRCC is insensitive to chemotherapy and radiation therapy. Surgical treatment is still the main treatment of ccRCC. However, more than 20% of kidney cancer patients relapse after undergoing nephrectomy. For kidney cancer patients with distant metastasis, the median survival time is about one year and the 5-year survival rate is less than 10% [3, 4]. Therefore, identifying new molecular markers as potential new prognostic biomarkers in ccRCC is particularly needed.

The TRIM family is a class of proteins with E3 ubiquitin ligase activity. Its members participate in many important biological processes, including autophagy, carcinogenesis, intracellular signal transduction, protein ubiquitination and degradation, and innate immunity. Recent studies have found that dysregulation of TRIM protein expression can cause a variety of diseases, such as tumors, neuropsychiatric diseases, immune diseases, chromosomal abnormalities, and developmental diseases [5]. The TRIM33 protein is a member of the TRIM family of proteins. The family has three conserved domains, from the N-terminus to the C-terminus, a zinc finger structure, one or two B-boxes, and a coiled-coil domain [6]. TRIM33 has been shown to participate in many biological processes, such as hematopoietic differentiation, embryonic development, cell cycle regulation, DNA repair, and immune response [7, 8]. In fact, TRIM33 has been suggested as a potential prognostic marker and therapeutic target for cancer patients. Cancer suppressive drugs targeting TRIM33 have been tested in various cancer mouse models and have shown good results [9, 10]. Studies have shown that there is a relationship between TRIM33 and the TGF-*β*/Smad pathway. TRIM33 has been identified as an effective regulator of TGF-*β*, which plays a positive or negative regulatory role in this pathway [11]. In renal clear cell carcinoma, studies have shown that TRIM33 is a direct target of miR-629 and affects tumor progression by inhibiting the TGF-*β*/Smad pathway [12]. However, other studies have shown that TRIM33 can influence the occurrence and development of glioblastoma through the Wnt/*β*-catenin pathway [13].

Unlike previous studies of renal clear cell carcinoma, for the first time, it was found that upregulation of TRIM33 inhibited the expression of *β*-catenin, cyclin D1, and c-myc molecules in the Wnt signaling pathway. In other words, TRIM33 can inhibit the progress of ccRCC by inhibiting the activity of the Wnt/*β*-catenin pathway. This is very important for elucidating the function of TRIM33 in ccRCC. TRIM33 plays a role as a tumor suppressor gene in ccRCC and is expected to become a new ccRCC therapeutic target and prognostic marker.

## 2. Materials and Methods

### 2.1. ccRCC Tissue Samples

With the patients' informed written consent, we collected 80 samples of human ccRCC tissues and normal kidney tissues from the Shandong Provincial Hospital during the period between 2013 and 2018. The pathological samples were first fixed with the corresponding fixative, then embedded in paraffin, and sectioned for immunohistochemical detection. The study was approved by the ethics committee of the Shandong Provincial Hospital. The principles of the Declaration of Helsinki and that of the World Medical Association were followed to conduct the study.

### 2.2. Cell Lines, Antibodies, and Drug

The cell line used in this study was purchased from the Chinese Academy of Sciences cell bank. The antibodies being used included rabbit anti-TRIM33 (Abcam, 47062 and 84455), anti-*β*-actin (Abcam, 8227), anti-E-cadherin (Abcam, 15148), anti-N-cadherin (Abcam, 18203), anti-vimentin (Abcam, 92547), anti-*β*-catenin (Abcam, 16051), anti-cyclin D1 (Abcam, ab40754), and anti-c-myc (Abcam, 32072) antibodies. Wnt agonist 1 was purchased from Selleck Chemicals (Shanghai, China) and was used at a final concentration of 10 *μ*M at 37°C for 24 h.

### 2.3. Bioinformatics Analysis

UALCAN, Human Protein Atlas, and Gene Expression Profiling Interactive Analysis (GEPIA) are three online analysis websites based on the TCGA database [14–16]. We explored the expression of TRIM33 in pan-cancer and its correlation with the clinical-pathological features of kidney cancer patients through these three online tools. Then, we downloaded the mRNA expression data of the KIRC part from the TCGA database. Using the drawing analysis software package in R language, we drew a scatterplot showing the expression of TRIM33. It contains 72 normal samples and 539 tumor samples.

### 2.4. Immunohistochemistry

First, the paraffin-embedded tissue specimens were sectioned and then subjected to routine deparaffinization and gradient alcohol processing. Then, we used citrate buffer (pH 6.0) for antigen retrieval. Afterward, the specimen on the section was incubated with anti-TRIM33 antibody at 4°C overnight. On the second day, the specimen on the section was incubated with the secondary antibody for 30 minutes in a 37°C incubator, and the DAB kit (Abcam) was used to detect the expression of the target protein and hematoxylin (Abcam) was used to stain the nucleus. Finally, two independent pathologists evaluated and calculated the corresponding *H* score.

### 2.5. Quantitative Real-Time PCR Assay

Total RNA was extracted with the TRIzol Reagent (Takara). Then, cDNA was synthesized from 2 *μ*g RNA using the Quantscript RT Kit (Takara). The reaction conditions for qRT-PCR were predenaturation at 94°C for 5 min, 94°C for 45 s, and 60°C for 60 s, for a total of 40 cycles. Three replicate wells were set for each sample. The primer sequences used for real-time PCR were as follows: TRIM33 forward, 5′-TGGACCAAAGGAAATGTGAACG-3′ and TRIM33 reverse, 5′-TGTGTGTCTGCATAAACTTGAACA-3′.

### 2.6. Western Blot Assay

First, we extracted total protein from the cell line, followed by protein denaturation, electrophoresis, membrane transfer, and incubation with primary antibody overnight in a shaker at 4°C. The next day, we incubated the Polyvinylidene Fluoride (PVDF) membrane with the secondary antibody for 1 hour at room temperature, and finally, we developed it according to the instructions of an ultrasensitive chemiluminescence kit (Millipore).

### 2.7. Establishment of TRIM33-Overexpressing Cells

To overexpress TRIM33 in kidney cancer cell lines, we commissioned OBiO Technology (Shanghai, China) to design a TRIM33 lentiviral vector for the expression of TRIM33 and an eGFP control lentiviral vector. We inoculated human kidney cancer cells into a 24-well plate. When the cells reached 40-50% confluency, a certain amount of lentivirus with polybrene (5 *μ*g) was added to the medium/ml. After 12 hours, the old medium was replaced with freshly completed medium, followed by incubation with 2 *μ*g/ml puromycin (Sigma-Aldrich) to select kidney cancer cell lines stably expressing TRIM33. After 72 hours, real-time PCR and western blotting were used to evaluate the transfection efficiency, thereby establishing two kidney cancer cell lines with stable TRIM33 overexpression.

### 2.8. Cell Counting Kit-8 Assay

The cells in both the control group and the experimental group were inoculated into 96-well plates at a density of 5,000 cells/well, and each group was provided with 3 double wells. After incubating the cells for a certain period of time, 10 *μ*l of Cell Counting Kit-8 (Dojindo) solution was added to each well. After incubating at 37°C for 1 h, absorbance was measured at 450 nm using a microplate reader.

### 2.9. Colony Formation Assay

Kidney cancer cells were seeded in 6-well plates at a density of 500 cells/well after incubating for 14 days at 37°C, fixing with 4% paraformaldehyde for 30 minutes and staining with 0.1% crystal violet for 10 minutes at room temperature. Image-Pro Plus software was used to count the colonies.

### 2.10. Wound Healing Assay

Kidney cancer cells were seeded in a 6-well plate, and when confluency reached about 95%, the cell layer was scraped using a 200 *μ*l sterile pipette tip. Then, the cell layer was washed thrice with PBS, and 2 ml of serum-free culture medium was added. Pictures were taken at certain times. The wound healing rate was evaluated using Image-Pro Plus.

### 2.11. Transwell Migration and Invasion Assays

We mixed the melted Matrigel gel (Corning) with serum-free medium according to a certain ratio (1 : 6), spread it evenly on the bottom of the Transwell chamber (upper chamber surface), and placed it in a 37°C incubator for coagulation for about 2 to 4 hours to make it appear gel-like. Subsequently, the cells that grew to the logarithmic phase were digested, counted, and the serum-free medium was diluted to a certain ratio and evenly inoculated to the bottom of the Transwell chamber, and then the Transwell chamber was cultured in a well plate with 10% FBS medium. After culturing the cells for 24 hours, we took out the Transwell chamber, wiped the cells inside the chamber and the remaining Matrigel glue with a cotton swab, washed it 3 times with PBS, and fixed the cells passing through the bottom and back of the chamber with paraformaldehyde, stained them with crystal violet, counted and analyzed them, and then drawn the corresponding histogram. The only difference between the cell migration experiment and the invasion experiment is that Matrigel is not used, and the rest of the steps are exactly the same.

### 2.12. Xenografts

First, we use Matrigel to make ACHN cells in logarithmic growth into a cell suspension with a concentration of 5 × 10^7^/ml. Subsequently, 100 *μ*l of cell suspension was injected into the left armpit of nude mice in the form of a subcutaneous injection. Then, we payed close attention and measured the changes in tumor volume every three days. Finally, at 35 days of rearing, the mice were sacrificed, and the tumors were excised for corresponding evaluation.

### 2.13. Statistics

The data are expressed as the mean ± S.E.M. Statistical analysis was performed using Prism software (GraphPad 8.0). The relationship between immunohistochemical staining and pathological parameters was determined by the Pearson *χ*^2^ test. Statistical significance of differences between groups was assessed using a two-tailed *t*-test and one-way ANOVA, respectively. A value of *P* < 0.05 was considered to indicate statistical significance.

## 3. Results

### 3.1. TRIM33 Expression in Pan-Cancer and Its Correlation with Clinical Features of Renal Cell Carcinoma Patients

**Figure 1:**
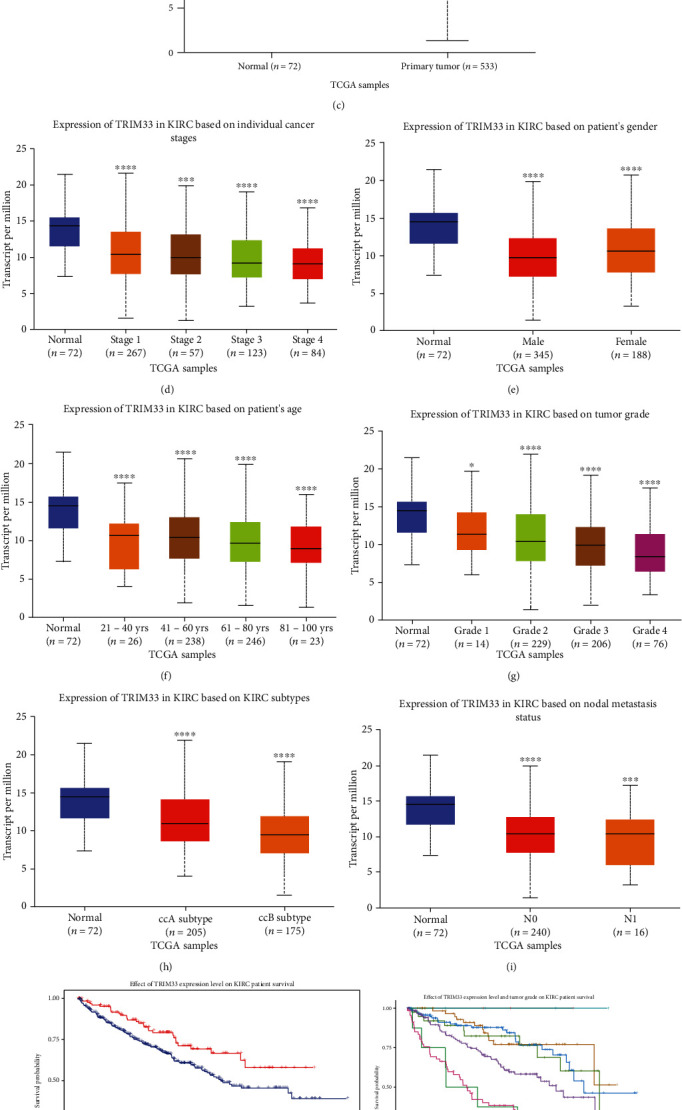
TRIM33 is downregulated in various tumors and is related to the progress of ccRCC. (a and b) TRIM33 expression in pan-cancer; red represents tumor tissue and blue represents normal tissue. (c) Analysis of differences in TRIM33 mRNA levels between ccRCC tissues and normal tissues through the UALCAN website based on TCGA. (d–i) The difference in expression of TRIM33 according to individual cancer stages, patient's gender, patient's age, tumor grade, KIRC subtypes, and nodal metastasis status. (j) Relationship between TRIM33 expression and survival of renal cell carcinoma patients. (k) KM plot depicting the association of TRIM33 expression levels and tumor grade with patient survival. (l) KM plot depicting the association of TRIM33 expression levels and gender with patient survival. (m and n) The relationship between the expression of TRIM33 in ccRCC and the survival of patients through the HPA website. ^∗^*P* < 0.05; ^∗∗^*P* < 0.01; ^∗∗∗^*P* < 0.001; ^∗∗∗∗^*P* < 0.0001.

**Figure 2:**
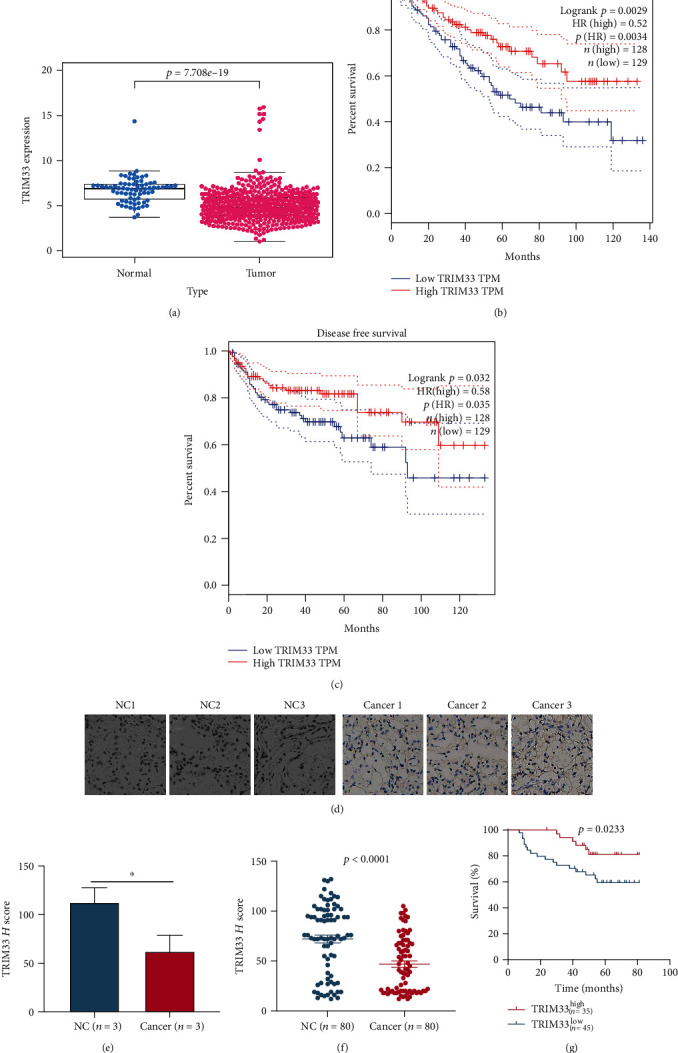
The expression of TRIM33 in kidney cancer tissues and normal tissues, and its relationship with the prognosis. (a) The expression of TRIM33 in kidney cancer tissues and normal tissues through the ccRCC data of the TCGA database; blue represents normal tissue and red represents tumor tissue, *P* = 7.708*e*‐19. (b and c) The relationship between OS, DFS, and TRIM33 expression in renal cell carcinoma patients. The cutoff value is 75%. (d and e) Immunohistochemical pictures of three typical kidney cancer tissues and normal tissues, and their *H* score. ^∗^*P* < 0.05. (f) Analysis of immunohistochemistry results of 80 cases of kidney cancer tissues and normal tissues. *P* < 0.0001. (g) The survival curve is drawn according to TRIM33 expression. *P* = 0.0233.

In order to understand the expression of TRIM33 in pan-cancer and renal cell carcinoma and the relationship between its expression and clinical characteristics, we used the online tool on UALCAN's website. We observed that TRIM33 is downregulated not only in KIRC but also in many cancers such as GBM, KIRP, THYM, and UCEC (Figures 1(a) and 1(b)). The expression of TRIM33 in KIRC was correlated with various clinical features like individual cancer stages, patient's gender, patient's age, tumor grade, KIRC subtypes, and nodal metastasis status (Figures 1(c)–1(i)). There was a correlation between the expression of TRIM33 and the survival of KIRC patients (*P* = 0.0084) (Figure 1(j)). Furthermore, it was found that the combination of the expression of TRIM33 and the patient's tumor grade correlated with the prognosis of the patient (*P* < 0.0001) (Figure 1(k)). Similarly, the combination of the expression of TRIM33 and the gender of the patient correlated with the prognosis of the patient (*P* = 0.042) (Figure 1(l)). Then, through the HPA website, we found that there was statistical significance between the expression of TRIM33 and KIRC patients (*P* = 0.0036) (Figures 1(m) and 1(n)). We also downloaded the data package of KIRC mRNA in TCGA and plotted the scatterplot of TRIM33 expression in KIRC using R language (Figure 2(a)). It was observed that the expression of TRIM33 in tumor tissue was significantly lower than that in the normal tissue (*P* = 7.708*e*‐19). Finally, the overall survival (*P* = 0.0034) curve and disease-free survival curve (*P* = 0.035) of TRIM33 in KIRC, which was drawn through the use of GEPIA website, also showed statistical significance (Figures 2(b) and 2(c)).

### 3.2. Immunohistochemistry Verified TRIM33 Expression in Renal Cell Carcinoma and Its Relationship with Survival of Renal Cell Carcinoma Patients

To examine the expression of TRIM33 in clinical tissues, we performed immunohistochemistry. Pictures of three typical samples are shown (Figure 2(d)). A histogram of the *H* score of these three typical samples (Figure 2(e)) and the scatter plot of the *H* score of these 80 samples (*P* < 0.0001) (Figure 2(f)) were drawn. Using our follow-up data on these 80 patients, we plotted the survival difference between the two groups with high expression and low expression of TRIM33, respectively. The survival of the group with low TRIM33 expression was significantly worse than the group with high TRIM33 expression (*P* = 0.0233) (Figure 2(g)). In addition, we found that there was a correlation between the expression levels of TRIM33 in renal cell carcinoma and two clinical features, tumor size (*P* = 0.031) and Furman's grade (*P* = 0.036) (Table 1).

### 3.3. Selection and Establishment of Renal Cancer Cell Lines Overexpressing TRIM33

**Figure 3:**
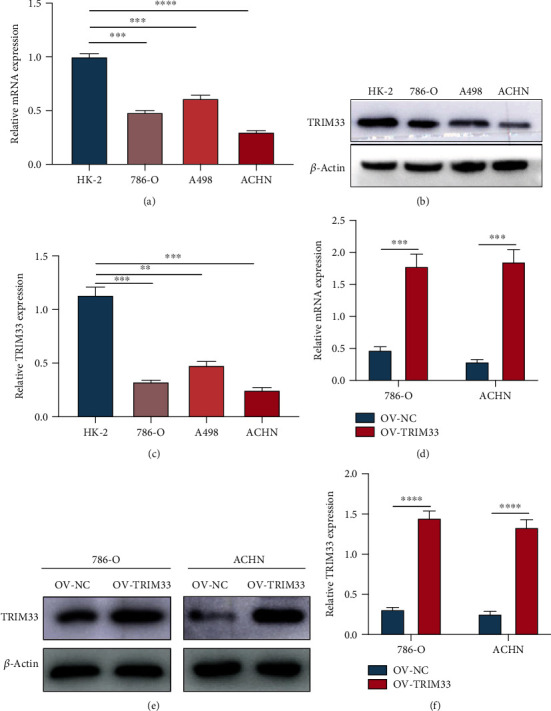
The expression of TRIM33 in HK-2, 786-O, A498, and ACHN cell lines and verification of the overexpression effect. (a) Quantitative real-time PCR was used to detect the expression of TRIM33 in HK-2, 786-O, A498, and ACHN cell lines. (b and c) Western blot analysis of the expression of TRIM33 in HK-2, 786-O, A498, and ACHN cell lines and data analysis. (d) Quantitative real-time PCR was used to verify TRIM33 overexpression in two kidney cancer cell lines. (e and f) Western blot analysis confirming TRIM33 overexpression in two kidney cancer cell lines and data analysis. ^∗∗^*P* < 0.01; ^∗∗∗^*P* < 0.001; ^∗∗∗∗^*P* < 0.0001.

In order to select the renal cancer cell lines suitable for this study, we used qRT-PCR and western blotting to determine the expression of TRIM33 in HK-2 and the three renal cancer cell lines. It was observed that the expression of TRIM33 in the three kidney cancer cell lines was significantly lower than that in normal renal tubular epithelial cells. Since the expression of TRIM33 in 786-O and ACHN cell lines was lower than that in the A498 cell line, we chose 786-O and ACHN cell lines for further experiments (Figures 3(a)–3(c)). In order to examine the effect of TRIM33, we used the lentiviral transfection technology to establish two kidney cancer cell lines that overexpress TRIM33, and detected the efficiency of overexpression by qRT-PCR and western blotting (Figures 3(d)–3(f)).

### 3.4. TRIM33 Overexpression Inhibits the Proliferation of 786-O and ACHN Cell Lines

**Figure 4:**
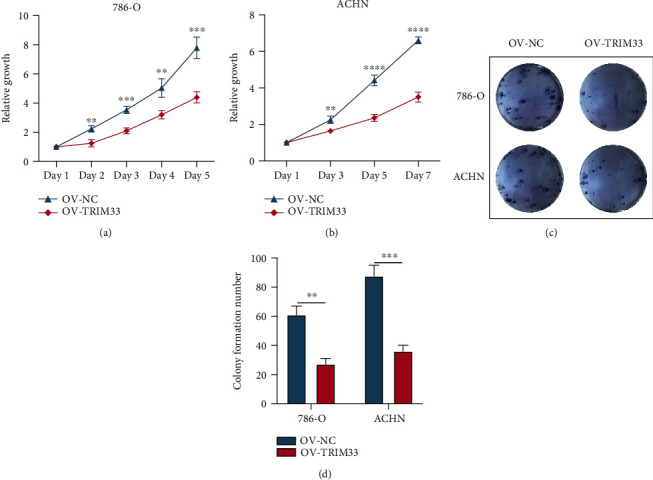
Cell proliferation experiment in vitro. (a and b) CCK-8 assay. The OD values of the OV-TRIM33 group and the OV-NC group in 786-O cells and ACHN cells were measured by the CCK-8 assay, and the relative growth rate of each group was evaluated. (c and d) Colony formation assay. Image-Pro Plus software was used to calculate the number of colonies formed. ^∗∗^*P* < 0.01; ^∗∗∗^*P* < 0.001; ^∗∗∗∗^*P* < 0.0001.

To investigate whether TRIM33 overexpression affects the proliferation of renal cancer cell lines, we used the CCK-8 assay (Figures 4(a) and 4(b)) and performed colony formation experiments (Figures 4(c) and 4(d)) to observe the changes between the overexpression group and the control group. We found that the proliferation of renal cancer cell lines overexpressing TRIM33 was decreased.

### 3.5. TRIM33 Overexpression Inhibits the Migration, Invasion, and EMT of 786-O and ACHN Cell Lines by Affecting the Activity of the Wnt Signaling Pathway

**Figure 5:**
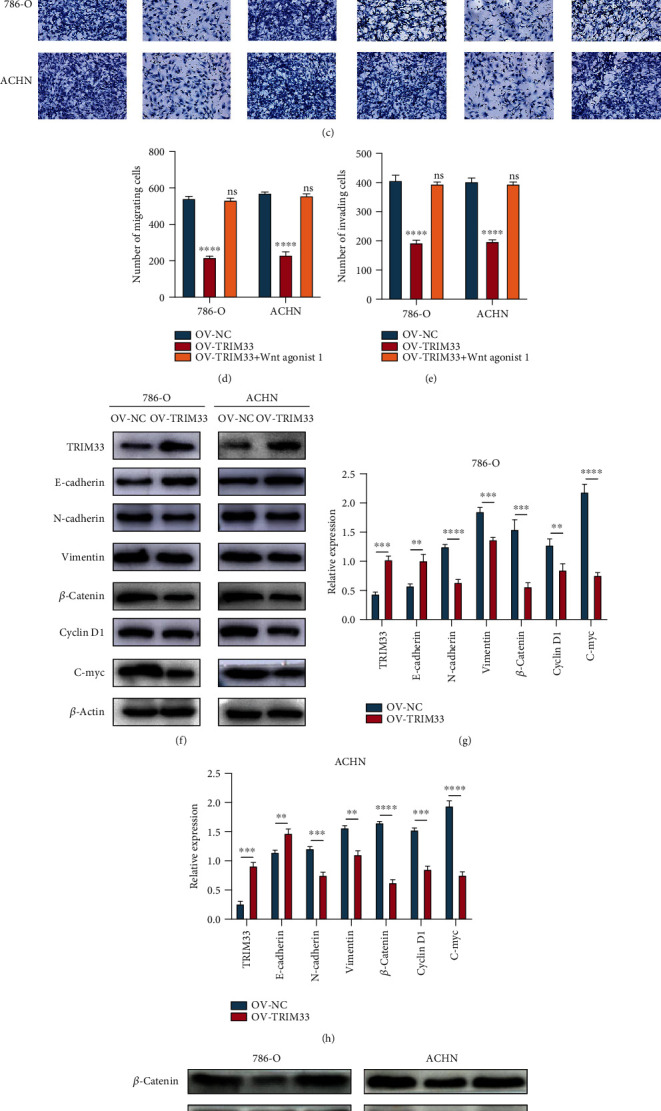
TRIM33 overexpression can inhibit migration, invasion, and EMT in ccRCC; here, the potential molecular mechanisms are explored. (a and b) Wound healing assay. (c–e) Transwell assay, including migration and invasion experiments. (f–h) Western blot assay for TRIM33, E-cadherin, N-cadherin, vimentin, *β*-catenin, cyclin D1, c-myc, and *β*-actin. (i–k) Western blot assay after incubation with a Wnt signaling pathway agonist for *β*-catenin, cyclin D1, c-myc, and *β*-actin. ^∗∗^*P* < 0.01; ^∗∗∗^*P* < 0.001; ^∗∗∗∗^*P* < 0.0001; ns represents no statistical significance.

Based on the effects of TRIM33 on the proliferation of kidney cancer cells, we conducted a series of experiments to investigate whether TRIM33 overexpression affects the migration and invasion of two kidney cancer cell lines. At the same time, we conducted a rescue experiment using an agonist of the Wnt signaling pathway. We used the wound healing (Figures 5(a) and 5(b)) and Transwell (Figures 5(c)–5(e)) assays to explore the differences between the three groups of OV-NC, OV-TRIM33, and OV-TRIM33+Wnt agonist 1. We found that the migration and invasion abilities of the two kidney cancer cell lines overexpressing TRIM33 were significantly inhibited. However, the addition of Wnt agonist 1 can effectively eliminate the inhibitory effect on the migration and invasion of the two renal cancer cell lines due to the overexpression of TRIM33. Through western blot experiments, we found that TRIM33 overexpression in two kidney cancer cell lines resulted in the upregulation of the expression of E-cadherin and downregulation of the expression of N-cadherin and vimentin. This indicates that TRIM33 overexpression in these two cell lines can inhibit epithelial-mesenchymal transition (EMT). At the same time, TRIM33 overexpression inhibited the expression of *β*-catenin, cyclin D1, and c-myc protein (Figures 5(f)–5(h)). To verify that TRIM33 exerts its inhibitory effect through the Wnt signaling pathway, we conducted a rescue experiment using the Wnt agonist 1. In the presence of the Wnt agonist 1, the originally downregulated *β*-catenin, cyclin D1, and c-myc protein expression levels were restored (Figures 5(i)–5(k)). This indicates that TRIM33 exerts its antitumor effect by inhibiting the activity of the Wnt/*β*-catenin pathway.

### 3.6. TRIM33 Overexpression Inhibits the Growth of the ACHN Cell Line in Mice

**Figure 6:**
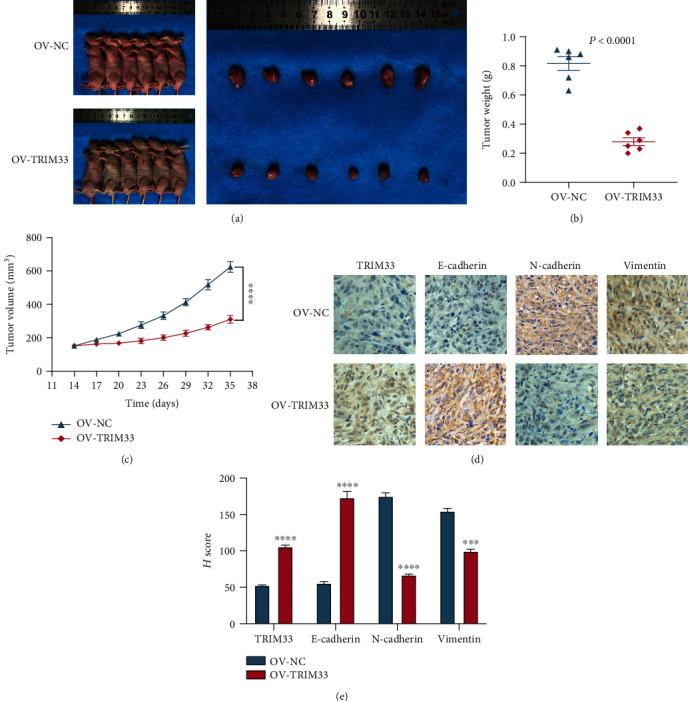
TRIM33 overexpression can inhibit tumor growth in vivo. (a) Pictures of nude mice and tumors. (b) Scatter plot of tumor weight between the OV-TRIM33 group and the OV-NC group. *P* < 0.0001. (c) Tumor volume measured every 3 days after the second week. (d, e) Immunohistochemistry staining for TRIM33, E-cadherin, N-cadherin, and vimentin. A histogram is used to display semiquantitative analysis results. ^∗∗∗^*P* < 0.001; ^∗∗∗∗^*P* < 0.0001.

**Figure 7:**
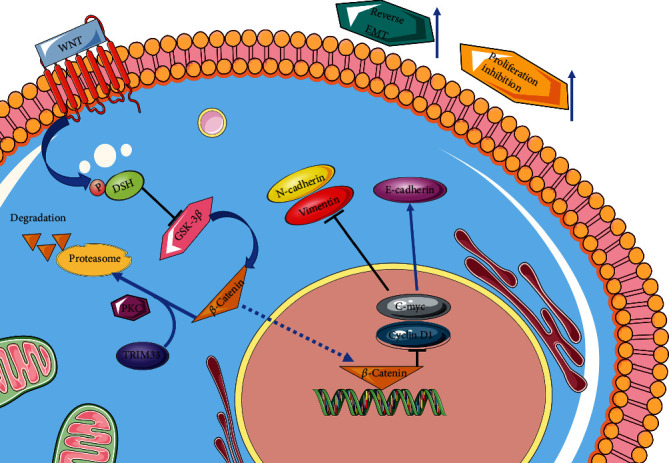
Schematic diagram of TRIM33 affecting renal cancer cell proliferation and EMT through the Wnt/*β*-catenin pathway (requires further experimental proof).

To investigate whether TRIM33 overexpression in renal cancer cell lines can inhibit tumor development in vivo, we conducted experiments in mice with subcutaneous tumors (Figure 6(a)). We chose the ACHN cell line as the cell line for animal experiments. We found that the tumor volume and weight in the TRIM33 overexpression group were significantly lower than those in the control group (Figures 6(b) and 6(c)). Then, we performed immunohistochemical analysis on the mouse tumor tissue. We found that the expression levels of TRIM33 and E-cadherin in tumor tissues in the TRIM33 overexpression group were significantly higher than those in the control group. The expression of N-cadherin and vimentin in the tumor tissue in the control group were significantly higher than that in the TRIM33 overexpression group (Figures 6(d) and 6(e)). From this experiment, we learned that TRIM33 plays a role in inhibiting the development of renal clear cell carcinoma both in vivo and in vitro. In particular, we also plotted the potential mechanism of TRIM33 in renal clear cell carcinoma cells (Figure 7).

## 4. Discussion

Clear cell renal cell carcinoma (ccRCC) is one of the most common malignant tumors of the urogenital system worldwide. Although a variety of treatments are now available to treat ccRCC, the global mortality rate of ccRCC has remained high in the past few decades. A large number of existing studies have confirmed that the occurrence and swelling of ccRCC is the result of the accumulation of cellular and molecular aberrations, including abnormalities in epigenetics, transcriptomics, miRNA, proteomics, and metabolomics [17–19]. All these studies indicate that ccRCC has significant molecular heterogeneity. Genes like *PAX8* have been identified as biomarkers in ccRCC through gene function analysis [20]. At present, the molecular characteristics of ccRCC have not been elucidated. Revealing the underlying mechanisms of the etiology and pathogenesis of ccRCC may help in the development of advanced treatment methods and the identification of effective biomarkers for diagnosis and prognosis.

Tripartite motif-containing 33 (TRIM33) is a protein with multiple biological functions. It has a RING domain at the N-terminus, two B-boxes, and a coiled-coil domain [21]. TRIM33 may be involved in the regulation of various biological processes of vertebrate embryonic and adult hematopoiesis [22], DNA repair [23], mitosis [24], transcriptional extension [25], and carcinogenesis [26]. However, the function of TRIM33 in tumors is complex; it can act as both a tumor suppressor or tumor promoter. It plays a tumor suppressor role in breast cancer, non-small-cell lung cancer, clear cell renal cell carcinoma, and glioma [12, 13, 27, 28]. However, it prevents tumor cell apoptosis in pancreatic cancer, B lymphoblastic leukemia, and cervical cancer [24, 29, 30] and thus acts as a tumor-promoting factor. These studies indicate that TRIM33 may affect tumor progression through multiple biological pathways. Besides, studies have shown that TRIM33 can participate in the TGF-*β* signaling pathway by binding to phosphorylated SMAD2/3 or monoubiquitinated SMAD4 in hepatocellular carcinoma, human chronic myelomonocytic leukemia, and pancreatic cancer [9, 10, 26]. In addition, some studies have shown that its tumor suppressor function may not be related to SMAD4 [31]. This indicates that the TGF-*β* signaling pathway may not be the main pathway through which TRIM33 inhibits tumorigenesis. Previous studies have mainly focused on the TGF-*β* signaling pathway, but now more attention is being paid to the Wnt signaling pathway [12, 32]. Studies in mouse embryonic stem cells have shown that TRIM33 regulates the Wnt/*β*-catenin pathway, a process not related to its E3 ligase activity of its RING domain. In human glioblastoma, it has been shown that TRIM33-mediated *β*-catenin destabilization is mediated through a dual effect. On the one hand, TRIM33 can inhibit the activity of Wnt3a, and on the other hand, it can inhibit EGF-induced transactivation of *β*-catenin [13]. It should be pointed out that in glioblastoma, downregulation of TRIM33 has been shown to lead to the activation of the Wnt/*β*-catenin pathway, thereby promoting tumor cell proliferation and tumorigenesis. These results support the key role of TRIM33 in nuclear degradation of *β*-catenin in human glioblastoma. In addition, there are two review articles showing that TRIM33 plays a key role in the Wnt signaling pathway by degrading *β*-catenin [33, 34]. However, whether TRIM33 can affect tumor progression through the Wnt/*β*-catenin pathway in ccRCC is still unknown.

To explore the potential role of TRIM33 in clear cell carcinoma, we used the TCGA database to reveal the mRNA expression levels of TRIM33 in human ccRCC tissues and its clinical relevance. TRIM33 showed significantly lower expression in kidney cancer tissues than in normal tissues, and its expression levels were significantly associated with multiple clinical features. The use of bioinformatics to identify targets that may play a role in tumor progression is becoming more and more popular [35]. Subsequently, we used 80 collected kidney cancer tissues and multiple kidney cancer cell lines to evaluate the expression levels of TRIM33. The results showed that TRIM33 was significantly downregulated in kidney cancer tissues and cell lines, and its expression was correlated with tumor size and Furman's grade. Furthermore, we found a significant correlation between the survival and the expression levels of TRIM33 in these 80 patients. To further study the potential biological role of TRIM33 in ccRCC, we stably overexpressed TRIM33 in 786-O and ACHN cell lines. We found that TRIM33 overexpression inhibited cell migration and invasion in two kidney cancer cell lines. We also found that TRIM33 overexpression affected the expression of *β*-catenin, cyclin D1, and c-myc in the Wnt signaling pathway. At the same time, incubation with a Wnt signaling pathway agonist restored migration and invasion as well as the levels of *β*-catenin, cyclin D1, and c-myc. Finally, in the mouse xenograft model, ACHN renal cancer cells overexpressing TRIM33 produced significantly smaller tumors in mice. In summary, these findings indicate that TRIM33 overexpression can exhibit antitumor activity by inhibiting the Wnt/*β*-catenin pathway in renal clear cell carcinoma.

As we all know, the Wnt/*β*-catenin pathway plays an important role in regulating cell proliferation, differentiation, self-renewal, adhesion, and migration in tumors [36–40]. The Wnt/*β*-catenin pathway can affect tumor growth and metastasis by regulating the expression of downstream genes such as cyclin D1 and c-myc [41]. Abnormal activation of the Wnt/*β*-catenin pathway has been observed in many types of human tumors, and it is thought to promote tumor development. Inhibiting the Wnt/*β*-catenin pathway has become an effective way for a variety of tumor-targeted therapies. For example, in esophageal squamous cell carcinoma, silencing SALL4 has been shown to inhibit EMT by inhibiting the activation of the Wnt/*β*-catenin pathway, thereby inhibiting the survival, migration, invasion, and drug resistance of cancer cells in vitro, as well as the tumorigenic ability in vivo [42]. In hepatocellular carcinoma, downregulation of miRNA-610 expression can activate the Wnt/*β*-catenin pathway to promote cancer cell proliferation, cell cycle progression, and tumorigenicity in vivo [43]. In breast cancer, miRNA-1299 overexpression promotes the proliferation and tumorigenicity of cancer cells by activating the Wnt/*β*-catenin pathway [44]. In cervical cancer, silencing DAX1 can significantly inhibit the proliferation of cancer cells in vitro, the properties of cancer stem cells and the tumorigenic ability in vivo, and this effect has also been shown to be related to the inhibition of Wnt/*β*-catenin pathway activation [45]. In renal clear cell carcinoma, when LGK974 was used to inhibit the expression of PORCN, the activity of the Wnt/*β*-catenin pathway was downregulated and proliferation, migration, and invasion were inhibited [46]. Similarly, our study shows that TRIM33 can inhibit the progression of renal clear cell carcinoma by inhibiting the activity of the Wnt/*β*-catenin pathway. This is important for understanding the role of the Wnt/*β*-catenin pathway in cancer.

## 5. Conclusions

In this study, we found that TRIM33 expression is reduced in ccRCC tissues and cells and is related to the poor prognosis of ccRCC. In vitro, TRIM33 overexpression inhibited the proliferation, colony formation, migration, invasion, and EMT ability of renal cancer cells. In vivo, the growth rate of TRIM33-overexpressing renal cancer cells was significantly inhibited. This may be due to the inhibition of the activity of the Wnt/*β*-catenin pathway by TRIM33. The limitation of this study is that we did not perform an in-depth analysis of the regulatory mechanism of TRIM33 on the Wnt/*β*-catenin pathway. Our study indicates that TRIM33 plays a role as a tumor suppressor gene in ccRCC and may become a potential target and prognostic marker for kidney cancer treatment in the future.

## Figures and Tables

**Table 1:** Association between TRIM33 levels in ccRCC tissues and clinicopathological features (*n* = 80).

Features	Number	TRIM33 expression		*χ* ^2^	*P*
		High (*n* = 35)	Low (*n* = 45)		
Gender					
Male	63	29	34	0.627	0.428
Female	17	6	11		
Age					
≤50	23	10	13	0.001	0.975
>50	57	25	32		
Location side					
Left	34	15	19	0.003	0.955
Right	46	20	26		
Tumor size (cm)					
≤4.0	44	24	20	4.630	0.031^∗^
>4.0	36	11	25		
Furman's grade					
G1+G2	62	31	31	4.374	0.036^∗^
G3+G4	18	4	14		
TNM stage					
I+II	77	35	42	2.424	0.119
III+IV	3	0	3		

Analysis was carried out with Fisher's exact *t*-test. The symbol ^∗^ indicates statistical significance (*p* < 0.05).

## Data Availability

The data used to support the findings of this study are available from the corresponding author upon request.

## Abbreviations

RCC: Renal cell carcinoma
ccRCC: Clear cell renal cell carcinoma
TRIM33: Tripartite motif-containing 33
KIRC: Kidney renal clear cell carcinoma
GBM: Glioblastoma multiforme
KIRP: Kidney renal papillary cell carcinoma
THYM: Thymoma
UCEC: Uterine corpus endometrial carcinoma
HPA: Human protein atlas
GEPIA: Gene expression profiling interactive analysis
TCGA: The Cancer Genome Atlas
EMT: Epithelial-mesenchymal transition.
